# Facial Appearance and Markers of Cardiometabolic Risk in Healthy Adult Men

**DOI:** 10.1007/s10508-025-03205-3

**Published:** 2025-08-20

**Authors:** Wioletta Czernicka, Agnieszka Żelaźniewicz, Judyta Nowak-Kornicka, Bogusław Pawłowski

**Affiliations:** Department of Human Biology, University of Wrocław, Wrocław, 51-148 Poland

**Keywords:** Lipid profile, Glycemia, Liver enzymes, Perceived attractiveness, Facial masculinity, Physical attractiveness, Cardiometabolic risk

## Abstract

Facial appearance is hypothesized to serve as a cue to an individual’s biological condition. However, the results of studies linking facial appearance with various measures of biological condition are inconclusive. Furthermore, most research has assessed the biological condition based on self-reported health or only a single health measure. This study aimed to verify if a man’s facial appearance reflects his metabolic health, which was assessed based on a broad panel of physiological biomarkers. The study included 105 healthy men with a mean age of 35.42 years (SD_age_ = 3.57). Cardiometabolic health was assessed based on lipid profile, inflammation, homocysteine levels, glycemia, and liver enzyme levels. Participants’ facial attractiveness and masculinity were assessed from photographs in an online survey. Facial attractiveness and masculinity were negatively related to homeostatic model assessment of insulin resistance index (HOMA-IR), triglycerides levels, and cardiometabolic risk score. However, these relationships were no longer significant after controlling for age, adiposity, and testosterone levels. Additionally, perceived facial masculinity and attractiveness were positively associated with testosterone levels and negatively with adiposity. Facial masculinity also showed a positive correlation with age. These findings align with previous research, which showed that men’s facial appearance conveys information about age, adiposity, and testosterone level but not about cardiometabolic health.

**Supplementary Information:**

The online version contains supplementary material available at 10.1007/s10508-025-03205-3.

## Introduction

From an adaptive perspective, physical attractiveness is thought to act as a cue of an individual’s biological condition guiding mate choice (Rhodes, 2006; Thornhill & Gangestad, 2006). Choosing an attractive partner may enhance an individual’s reproductive success by increasing the likelihood of conceiving healthy offspring. Research shows that facial appearance is the key predictor of overall attractiveness, possibly because it is more salient than body shape (Peters et al., 2007; Rhodes et al., 2005). Traits such as pronounced facial sexual dimorphism (Yang et al., 2015), symmetry (Hönekopp et al., 2004), and lower adiposity (Coetzee et al., 2009; De Jager et al., 2018) are expected to develop and be displayed only in individuals with good health and high fertility. These traits are also hypothesized to correlate with various measures of biological condition (Henderson et al., 2016; Zahavi, 1975).

However, the results of the studies linking facial appearance with an individual’s biological condition have yielded inconclusive results (Jones et al., 2021, 2022). Some findings support a positive relationship between perceived facial attractiveness and immunity, including stronger antibody response after vaccination (Rantala et al., 2012), heightened cytokine response (Phalane et al., 2017), increased phagocytosis of *E. coli*, higher basophil counts, lower neutrophil counts, greater NK cell cytotoxicity, and slower rates of *S. aureus* growth in plasma (Mengelkoch et al., 2022) or fewer infections per year (Gray & Boothroyd, 2012). Additionally, facial attractiveness has been associated with fewer past health problems (Hume & Montgomerie, 2001), higher fertility (Roberts et al., 2004; Soler et al., 2014; Żelaźniewicz et al., 2021), and lower oxidative stress level (Gangestad et al., 2010). However, other studies failed to show any relationship between perceived facial attractiveness and health (Coetzee et al., 2007), including immunoglobulin A level (Cai et al., 2019), post-vaccination response (Pátková et al., 2022; Rantala et al., 2013), or oxidative stress level (Foo et al., 2017). Similarly, research examining the biological correlates of specific facial traits central to attractiveness has produced mixed results. For instance, morphological masculinity has been positively associated with the number of sexual partners (Polo et al., 2019), and is strongly preferred by women who perceive themselves as attractive (Docherty et al., 2020). Although morphological masculinity has been positively associated with the number of sexual partners (Polo et al., 2019) and is strongly preferred by women who perceive themselves as attractive (Docherty et al., 2020), its reliability as a marker of male biological condition remains debated. Some studies suggest that facial masculinity is a reliable marker of a man’s biological condition (Foo et al., 2020; Phalane et al., 2017; Rhodes et al., 2003; Thornhill & Gangestad, 2006), others indicate that it is not related to men's health (Boothroyd et al., 2005; Zaidi et al., 2019), or conclude that masculinity is, at best, a weak cue of immunity (Boothroyd et al., 2013).

Most research on attractiveness focuses on its relationship with perceived (rather than objectively measured) health (Henderson et al., 2016) or examines only specific aspects of health, such as immunity (Hume & Montgomerie, 2001; Phalane et al., 2017) or fertility (Roberts et al., 2004; Soler et al., 2014). However, only a few studies suggest that physical attractiveness may also serve as a cue to cardiometabolic homeostasis. Hansell et al. (1982) reported findings from four studies conducted on different samples of adolescents and older adults of both sexes, showing that perceived attractiveness, rated by both men and women, was negatively related to blood pressure, but only in adolescent women. Bulczak and Gugushvili (2023), using panel survey data from the National Longitudinal Study of Adolescent to Adult Health in the United States (Add Health), found that physical attractiveness, rated by an interviewer during in-person interview, was positively related with adult cardiometabolic health in both sexes, measured at 10-year follow-up. Cardiometabolic health was assessed based on a set of biomarkers: LDL cholesterol, glucose, C-reactive protein (CRP), blood pressure, and resting heart rate. Facial attractiveness was also linked to shorter cardiac recovery time in young adult men but not in women, although this study was based on a relatively small sample (N = 100) (Shackelford & Larsen, 1999). Finally, a cross-sectional study of healthy women aged 25–35 found that perceived attractiveness was negatively related to triglyceride levels (Żelaźniewicz et al., 2020a) and to leptin levels—a hormone synthesized by adipose tissue that, at higher levels, may negatively impact metabolic health (Żelaźniewicz et al., 2020b). Furthermore, research suggests that one of the key predictors of facial attractiveness is body and facial adiposity, i.e. traits that have a direct impact on cardiometabolic health (Coetzee et al., 2009; De Jager et al., 2018; Han et al., 2016; Rantala et al., 2013).

Cardiometabolic health is an important component of an individual’s biological condition and may be particularly important in men, as they are at higher risk of developing cardiometabolic syndrome (Dar, 2023; Slagter et al., 2017). Moreover, the abnormalities in the key aspects predicting cardiometabolic health, such as central obesity, insulin resistance, dyslipidemia, and hypertension, extend well beyond the cardiovascular system (Morrison & Brannigan, 2015). For instance, cholesterol, a component of cardiometabolic health, also plays a vital role in reproduction, as it serves as a substrate for sex hormones synthesis. Genetic polymorphisms that cause functional modifications in proteins involved in cholesterol uptake, mobilization, and de novo synthesis have been shown to significantly influence human fertility (DeAngelis et al., 2014). According to the antagonistic pleiotropy hypothesis, natural selection favors genes that enhance reproductive success, even if they have negative effects later in life, over those that promote longevity but offer fewer reproductive and survival benefits during the reproductive years (Byars & Voskarides, 2020). For instance, the APOE4 allele of the gene encoding apolipoprotein E, which is associated with higher cholesterol levels, has been positively linked to fertility in women from a natural fertility population of forager-horticulturists (Trumble et al., 2023). However, it is also associated with accelerated cellular aging (Jacobs et al., 2013) and an increased risk of mortality (Wolters et al., 2019). Interestingly, in men, genetic variants related with both lower and higher cholesterol level appears to be linked to reduced fertility (Gerdes et al., 1996). Furthermore, studies examining the relationship between cardiometabolic health and fertility generally indicate a positive correlation in adult men (Eisenberg et al., 2016; Gur et al., 2021). Metabolic syndrome is also considered one of the factors contributing to the global decline in male fertility (Mann et al., 2020). Blood pressure, lipid profile, and glycemia, for example, are negatively related with sperm normal morphology (Chen et al., 2020; Lotti et al., 2021; Zhao & Pang, 2020) as well as testosterone level (Ehala-Aleksejev & Punab, 2018; Hansen et al., 2023; Zhong et al., 2021; Hamad Zubi & Hamad Alfarisi, 2021). Additionally, metabolic syndrome is characterized by chronic systemic inflammation and increased oxidative stress, both of which are linked to numerous diseases (Monserrat-Mesquida et al., 2020). Metabolic syndrome or its components are also related to periodontitis (Abdalla-Aslan et al., 2019), and to an increased risk of colorectal cancer and non-melanoma skin cancers in both sex, as well as thyroid cancer in women (Lee et al., 2020; Nguyen et al., 2022; Rezaiian et al., 2022).

There is some evidence that components of cardiometabolic health may be reflected in facial appearance, which could impact attractiveness and mate choice. For instance, Hu et al. (2019) demonstrated that metabolic syndrome is associated with several skin conditions, such as psoriasis, *acne vulgaris*, *hidradenitis suppurativa*, androgenetic alopecia, *acanthosis nigricans*, and atopic dermatitis. Elevated glycemia may increase perceived facial age, as glucose metabolism is related to skin aging (Blazer et al., 2002; Dekker et al., 2009) and skin and hair abnormalities such as acrochordons, *acanthosis nigricans*, androgenetic alopecia, acne, and hirsutism (González-Saldivar et al., 2017). The relationship between facial appearance and lipid profile may be driven by its impact on current and future health and/or by its role as a substrate for sex hormone synthesis (Daniels et al., 2009). Furthermore, dyslipidemia is involved in the pathogenesis of primary cicatricial and androgenic alopecia or congenital hypertrichosis (Palmer et al., 2020). Bulczak and Gugushvili (2023) found a negative relationship between individuals' self-rated physical attractiveness and cardiometabolic risk. However, only one study has examined the direct relationship between female facial appearance and markers of metabolic health. This study found a negative correlation between facial attractiveness and lipid profile components detrimental to health (total cholesterol, LDL, triglycerides), but no correlation with relatively protective health markers such as HDL. Additionally, no relationship was found between women's facial appearance and glucose metabolism, liver function, or inflammatory markers. However, this study was conducted among relatively young women, a sex that is more protected against various metabolic health risks due to higher estradiol levels, especially in younger age (Żelaźniewicz et al., 2020a). Furthermore, Shackelford and Larsen (1999) demonstrated that attractive participants, compared to unattractive ones, exhibited better cardiovascular health. This effect was stronger for men than for women, suggesting that the relationship between metabolic health and facial appearance may be more apparent in men. To date, no studies have linked facial masculinity to cardiometabolic health markers, despite the fact that testosterone—the hormone primarily influencing masculinity—has significant implications for cardiometabolic health (Kaur & Werstuck, 2021).

Here, we tested if a man’s facial appearance (attractiveness and masculinity) is a cue to his metabolic health. In a sample of men aged 30–45 years, we examined the relationship between perceived facial attractiveness and masculinity and various metabolic health markers, including lipid profile, glycemia markers, liver enzymes, inflammation level, and cardiometabolic risk score. Since morphological and metabolic health variability increases with age, we focused on men over 30 but younger than 45 years—an age range before many cardiometabolic disorders typically develop. We hypothesized that perceived facial attractiveness and masculinity are negatively related to the levels of proatherogenic cholesterol, triglycerides, and LDL, while positively related to antiatherogenic HDL. Furthermore, we hypothesized, that glycated hemoglobin (HbA1C) and insulin resistance index (HOMAI-IR) negatively predict men’s perceived facial attractiveness and masculinity. Additionally, we hypothesized that the liver enzyme ratio, inflammation levels, and cardiometabolic risk score would also be negatively related to perceived facial attractiveness and masculinity. We controlled for age, adiposity, and testosterone level due to their potential impact on men’s facial attractiveness (He et al., 2021; De Jager et al,. 2018), facial masculinity (Penton-Voak & Chen, 2004), and metabolic health biomarkers (Kaur & Werstuck, 2021; Tam et al., 2020; Vekic et al., 2019).

## Method

### Participants

This study was part of a broader research project investigating predictors of male health, conducted on 209 men (M_age_ = 35.26, SD_age_ = 3.49) from a western, urban population. Participants were recruited by information in local media or social networks from a general population. Participants were informed about the purpose of the research and gave written consent to participate.

Participants were required to meet the following criteria: age between 30 and 45 years, no smoking, no hormonal treatment, no diagnosis with chronic diseases (e.g. diabetes, cardiovascular, or autoimmune diseases), and no ongoing infection. Current health status was assessed based on blood morphology with smear and CRP level. From the initial group of participants, 34 were excluded from the analyses due to the following reasons: (1) smoking (N = 8); (2) elevated CRP level (CRP > 10 µg/ml), indicating ongoing inflammatory state (N = 1); (3) chronic diseases (N = 12); (4) missing data (N = 13). As the initial analyses showed that having a beard impacted the participant’s perceived attractiveness and masculinity, bearded men were also excluded from the analyses (N = 70). Thus, the final analyses included 105 healthy men of age between 29.95 and 44.29 years (M_age_ = 35.39, SD_age_ = 3.58).

### Procedure

A fasting blood sample was taken between 7:30 a.m. and 9:00 a.m. for blood biochemical and hormone analyses. Participants were asked to refrain from physical activity, heavy meals, and alcohol consumption for 24 h before the study visit. The participants completed a questionnaire on demographic data, current and past health problems, medication use, physical activity, smoking, and alcohol consumption patterns.

Face photographs were taken with the same SLR digital camera (Nikon D7100 with Tamron SP AF 17–50 mm F/2.8 XR Di II LD IF camera lens), from the same distance and constant lighting conditions. Participants were asked to pose with a neutral facial expression, to remove glasses or piercings. A uniform white screen was used as a background. The photographs were then cropped into an oval shape to obscure information about hairstyles and clothing.

### Measures

#### Face Attractiveness and Masculinity Assessment: Online Survey

Perceived facial attractiveness and facial masculinity were assessed on photographs in an online survey by women aged between 26.74 and 47.74 years (M_age_ = 35.81, SD_age_ = 4.62) recruited online, via social media, internet forums, and university webpages. Informed consent for participation in the study was obtained from all women. Each face was assessed by a minimum of 55 women (every woman assessed 15 photos). The photographs were presented in random order. Women were asked to evaluate men’s facial attractiveness (“How attractive is the man in the photo?”) and masculinity (“How masculine is the man in the photo?”) on a scale from 1 (not at all attractive/masculine) to 9 (very attractive/masculine).

#### Anthropometry

Adiposity was measured with bioimpedance analysis (SECA mBCA 515). Waist circumference was measured twice at the midpoint between the lower border of the rib cage and the iliac crest (c.a. at the level of the umbilicus) with flexible tape. The mean of the two measurements was used in the analysis.

#### Hormone Assay

Quantitative measurements of total testosterone levels were assayed by a certified analytical laboratory (DIAGNOSTYKA®) using a Cobas analyzer and expressed in ng/dl.

#### Cardiometabolic Risk Indices

 + Morning blood pressure was measured twice in a seated position using a digital sphygmomanometer, following a standard protocol and the mean value was used in the analyses. Mean blood pressure (MBP) was calculated from the mean systolic (SBP) and mean diastolic (DBP) blood pressure according to the formula: MBP = 1/3 (SBP-DBP) + DBP.

The cardiometabolic risk score was calculated based on standardized values of waist circumference, MBP, glucose, HDL, and triglycerides levels, the clinical criteria for defining the risk of metabolic syndrome set by the International Diabetes Federation. To reflect increasing cardiometabolic risk with higher values, the standardized HDL values were multiplied by -1. Cardiometabolic risk scores were then calculated as the arithmetic mean of these five standardized values, with lower scores indicating a better cardiometabolic risk profile. Additionally, the cardiometabolic risk was estimated based on total cholesterol, homocysteine, and hsCRP levels.

The cholesterol-triglyceride index was obtained by calculation of z-scores for total cholesterol, HDL (multiplied by -1), LDL, and triglycerides, and then summing these values and dividing by 4.

Glucose, HDL, triglycerides, and total cholesterol levels were measured in serum at a certified laboratory (DIAGNOSTYKA) using spectrophotometry with a Cobas analyzer. Homocysteine levels were measured using the turbidimetric method with a Cobas analyzer at the same laboratory. hsCRP levels were determined using an enzyme-linked immunosorbent assay (ELISA) kit (DEMEDITEC® DE740011) in accordance with the manufacturer’s instructions. The inter- and intra-assay coefficients of variation were less than 6.3% and 6.9%, respectively, with an assay sensitivity of 0.02 µg/ml. The absorbance was read on an ASYS UVM340 spectrophotometer at λ = 450 nm. The standard curve was generated by plotting each standard's average absorbance value (y-axis) against its concentration (x-axis). hsCRP levels were calculated from the standard curve and expressed in µg/ml.

Insulin sensitivity was estimated based on glycated hemoglobin level, the homeostatic model assessment of insulin resistance index (HOMA-IR), which was calculated using the following formula: fasting insulin (mU/L) × fasting glucose (mmol/L)/22.5. Fasting insulin level was measured in serum according to the procedure IB/LAB/100 in a certified laboratory (DIAGNOSTYKA). Glycated hemoglobin (HbA1c) levels were determined in blood using the HPLC method with a Variant analyzer.

Liver function, related to cardiometabolic risk, was evaluated based on the AST/ALT ratio. ALT and AST levels were assayed in a certified laboratory (DIAGNOSTYKA). Creatinine levels were also assessed to monitor renal function, with measurements conducted at the same laboratory.

### Statistical Analyses

Based on visual inspection of the graphs, the results of the Shapiro–Wilk test, and the values of kurtosis and skewness, the distribution of triglycerides, total cholesterol, LDL cholesterol, homocysteine, HOMA-IR, ALT, AST, creatinine, testosterone levels, and the AST/ALT ratio were assessed as non-normally distributed. These variables were log-transformed, and in each case, the distribution became normal or near-normal, with skewness and kurtosis z-scores less than or equal to 3.29 (Kim, 2013).

The relationship between perceived facial attractiveness or masculinity and cardiometabolic risk markers levels was verified with Pearson correlation. Pearson correlation was also used to verify the relationships between controlled variables. A simple linear regression model was then employed to test the association between perceived facial attractiveness or masculinity and the levels of cardiometabolic risk markers, adjusting for age, adiposity, and testosterone levels. Due to collinearity among some predictors (see Table 3), we applied centering to the variables included in the regression model. The model fit was evaluated using the Akaike Information Criterion (AIC). Finally, mediation analyses were conducted using the medmod module in Jamovi (2.4.14) to assess possible mediating effects among the predictors. The significance of indirect effects was assessed using a bootstrap procedure with 5000 resamples.

Analyses were performed with Statistica 12.0 and Jamovi 2.4.14 software. The results were interpreted as statistically significant if *p* < 0.05.

## Results

### Descriptive Statistics

Mean values, ranges, and standard deviations of facial attractiveness and masculinity, health markers, age, and controlled variables are presented in Table 1.

**Table 1:** Descriptive statistics of the analyzed variables (N = 105)

	M	SD	Min	Max
Age [years]	35.39	3.58	29.95	44.29
Total cholesterol [mg/dl]	191.03	31.97	129.00	332.00
HDL [mg/dl]	54.12	11.62	29.00	88.00
LDL [mg/dl]	114.93	29.91	44.00	255.00
Triglycerides [mg/dl]	109.68	59.57	37.00	389.00
Cholesterol-triglyceride index	**− **0.02	0.58	**− **1.54	2.07
HbA1c [mmol/mol]	33.86	2.72	27.00	40.00
HOMA-IR	2.04	1.10	0.41	6.16
ALT [U/l]	25.49	12.38	8.00	67.00
AST [U/l]	23.60	7.25	11.00	50.00
AST/ALT	1.03	0.32	0.46	2.17
hsCRP [µg/ml]	1.10	1.21	0.01	6.49
Face attractiveness (1–9)	2.94	0.87	1.58	5.64
Face masculinity (1–9)	5.32	0.72	3.42	7.38
Adiposity [%]	22.16	6.28	6.07	37.07
Homocysteine [μmol/l]	13.46	2.72	8.80	23.43
Creatinine [mg/dl]	0.94	0.11	0.65	1.40
Cardiometabolic risk score	**− **0.06	0.60	**− **1.47	1.90
Total testosterone [ng/dl]	502.53	175.68	135.30	1010.00

### Facial Appearance and Cardiometabolic Risk Markers

Perceived facial masculinity was negatively related to adiposity, HOMA-IR, cardiometabolic risk score, and triglyceride levels. It was positively correlated with age, total testosterone level, ALT/AST ratio, HDL, and cholesterol level. Perceived facial attractiveness was negatively related to adiposity, HOMA-IR, cardiometabolic risk score, and triglycerides level. Moreover, there was a positive correlation between facial attractiveness and total testosterone level, ALT/AST ratio, and HDL cholesterol (Table 2; the plots are included in the supplementary materials).

**Table 2:** Correlation values for the relationship between biomarkers of health and face attractiveness and face masculinity (N = 105)

	Masculinity		Attractiveness	
	r	*p*	*r*	*p*
Age [years]	**0.35**	** < 0.001**	**− **0.08	0.42
Adiposity [%]	**− 0.26**	**0.01**	**− 0.32**	** < 0.001**
Total testosterone LOG [ng/dl]	**0.39**	** < 0.001**	**0.23**	**0.02**
HOMA-IR LOG	**− 0.20**	**0.04**	**− 0.25**	**0.01**
HbA1c [mmol/mol]	**− **0.11	0.27	**− **0.04	0.72
Cardiometabolic risk score	**− 0.20**	**0.04**	**− 0.33**	**0.001**
Creatinine [mg/dl]	**− **0.02	0.87	**− **0.01	0.90
AST/ALT LOG	**0.28**	**0.004**	**0.23**	**0.02**
Homocysteine LOG [μmol/l]	0.04	0.71	**− **0.03	0.78
Total cholesterol LOG [mg/dl]	**− **0.01	0.94	**− **0.10	0.29
HDL cholesterol [mg/dl]	**0.25**	**0.01**	**0.31**	**0.002**
LDL cholesterol LOG [mg/dl]	**− **0.26	0.79	**− **0.16	0.10
Triglycerides LOG [mg/dl]	**− 0.25**	**0.01**	**− 0.23**	**0.02**
Cholesterol-triglyceride index	**− **0.15	0.12	**− 0.25**	**0.01**

Bolded values are significant at *p* < 0.05

Total testosterone levels were negatively associated with HOMA-IR, cardiometabolic risk score, triglyceride levels, and the cholesterol-triglyceride index but positively associated with AST/ALT and HDL cholesterol. Adiposity was negatively correlated with creatinine, AST/ALT, and HDL cholesterol. At the same time, it was positively correlated with HOMA-IR, cardiometabolic risk score, total cholesterol, LDL cholesterol, triglycerides, and the cholesterol-triglyceride index. Age was not associated with cardiometabolic risk markers (Table 3). Additionally, perceived facial attractiveness was positively correlated with perceived facial masculinity (*r* = 0.52, *p* < 0.001).

**Table 3:** Correlation between total testosterone level, adiposity, age and, cardiometabolic risk markers (N = 105)

	Total testosterone LOG		Adiposity [%]		Age	
	*R*	*p*	*r*	*p*	*r*	*p*
HOMA-IR LOG	**− 0.32**	**0.001**	**0.57**	** < 0.001**	**− **0.04	0.70
HbA1c [mmol/mol]	**− **0.05	0.64	**− **0.01	0.95	0.07	0.49
Cardiometabolic risk score	**− 0.41**	** < 0.001**	**0.69**	** < 0.001**	0.00	0.99
Creatinine [mg/dl]	0.17	0.09	**− 0.25**	**0.01**	**− **0.00	0.98
AST/ALT LOG	**0.31**	**0.001**	**− 0.56**	** < 0.001**	0.07	0.46
Homocysteine LOG [μmol/l]	**− **0.06	0.50	0.05	0.63	0.06	0.52
Total cholesterol LOG [mg/dl]	**− **0.12	0.21	**0.21**	**0.04**	0.08	0.42
HDL cholesterol [mg/dl]	**0.36**	** < 0.001**	**− 0.50**	** < 0.001**	0.13	0.20
LDL cholesterol LOG [mg/dl]	**− **0.07	0.48	**0.22**	**0.02**	0.04	0.67
Triglycerides LOG [mg/dl]	**− 0.52**	** < 0.001**	**0.52**	** < 0.001**	**− **0.09	0.32
Cholesterol-triglyceride index	**− 0.33**	**0.001**	**0.48**	** < 0.001**	**− **0.01	0.94

Bolded values are significant at *p* < 0.05

Regression analysis revealed that perceived facial attractiveness was primarily predicted by adiposity and was not associated with any cardiometabolic risk markers (cholesterol-triglyceride index, cardiometabolic risk score, HOMA-IR, and ALT/AST log) when controlling for age and testosterone levels (Table 4). There was no relationship between perceived facial masculinity and any of the cardiometabolic risk markers when controlled for adiposity, age, and testosterone level. Perceived facial masculinity was only predicted by age and testosterone levels (Table 5).

**Table 4:** Results of regression analyses for the relationship between facial attractiveness and selected cardiometabolic risk markers controlled for covariates (N = 105)

	Estimate (β)	SE	t(100)	*p*
Model 1: Dependent variable: facial attractiveness: F(4,100) = 4.04, adj.R^2^ = 0.11, *p* = 0.004, f^2^ = 0.16				
Adiposity	**− 0.03**	**0.02**	**− 2.20**	**0.03**
Age	**− **0.03	0.02	**− **1.29	0.20
Testosterone LOG	0.27	0.24	1.09	0.28
Cholesterol-triglyceride index	**− **0.15	0.16	**− **0.94	0.35
Model 2: Dependent variable: facial attractiveness: F(4,100) = 3.82, adj.R^2^ = 0.10, *p* = 0.006, f^2^ = 0.15				
Adiposity	**− 0.04**	**0.02**	**− 2.45**	**0.02**
Age	**− **0.03	0.02	**− **1.36	0.18
Testosterone LOG	0.27	0.26	1.05	0.30
Triglycerides LOG	**− **0.07	0.23	**− **0.31	0.76
Model 3: Dependent variable: facial attractiveness: F(4,100) = 4.19, adj.R^2^ = 0.11, *p* = 0.004, f^2^ = 0.17				
Adiposity	**− **0.03	0.02	**− **1.43	0.16
Age	**− **0.03	0.02	**− **1.19	0.24
Testosterone LOG	0.24	0.24	1.00	0.32
Cardiometabolic risk score	**− **0.22	0.19	**− **1.18	0.24
Model 4: Dependent variable: facial attractiveness: F(4,100) = 3.93, adj.R^2^ = 0.10, *p* = 0.005, f^2^ = 0.16				
Adiposity	**− 0.03**	**0.02**	**− 2.06**	**0.04**
Age	**− **0.03	0.02	**− **1.32	0.19
Testosterone LOG	0.28	0.24	1.19	0.24
HOMA-IR LOG	**− **0.29	0.43	**− **0.69	0.50
Model 5: Dependent variable: facial attractiveness: F(4,100) = 3.87, adj.R^2^ = 0.10, *p* = 0.006, f^2^ = 0.15				
Adiposity	**− 0.04**	**0.02**	**− 2.17**	**0.03**
Age	**− **0.03	0.02	**− **1.35	0.18
Testosterone LOG	0.29	0.24	1.21	0.23
AST/ALT LOG	0.17	0.32	0.53	0.60

Bolded values are significant at *p* < 0.05

**Table 5:** Results of regression analyses for the relationship between facial masculinity and selected cardiometabolic risk markers controlled for covariates (N = 105)

	Estimate	SE	t(100)	*p*
Model 1: Dependent variable: facial masculinity: F(4,100) = 8.70, adj.R^2^ = 0.23, *p* < 0.001, f^2^ = 0.35				
Adiposity	**− **0.01	0.01	**− **0.85	0.40
Age	**0.06**	**0.02**	**3.50**	** < 0.001**
Testosterone LOG	**0.64**	**0.19**	**3.47**	** < 0.001**
Cholesterol-triglyceride index	**− **0.001	0.12	**− **0.01	0.99
Model 2: Dependent variable: facial masculinity: F(4,100) = 8.71, adj.R^2^ = 0.23, *p* < 0.001, f^2^ = 0.35				
Adiposity	**− **0.01	0.01	**− **0.82	0.41
Age	**0.06**	**0.02**	**3.50**	** < 0.001**
Testosterone LOG	**0.64**	**0.20**	**3.21**	**0.002**
Triglycerides LOG	**− **0.01	0.17	**− **0.08	0.94
Model 3: Dependent variable: facial masculinity: F(4,100) = 8.71, adj.R^2^ = 0.23, *p* < 0.001, f^2^ = 0.35				
Adiposity	**− **0.01	0.01	**− **0.68	0.50
Age	**0.06**	**0.02**	**3.48**	** < 0.001**
Testosterone LOG	**0.64**	**0.19**	**3.43**	** < 0.001**
Cardiometabolic risk score	**− **0.01	0.15	**− **0.07	0.95
Model 4: Dependent variable: facial masculinity: F(4,100) = 8.78, adj.R^2^ = 0.23, *p* < 0.001, f^2^ = 0.35				
Adiposity	**− **0.01	0.01	**− **0.55	0.57
Age	**0.06**	**0.02**	**3.53**	** < 0.001**
Testosterone LOG	**0.63**	**0.18**	**3.44**	** < 0.001**
HOMA-IR LOG	**− **0.16	0.33	**− **0.49	0.63
Model 5: Dependent variable: facial masculinity: F(4,100) = 9.50, adj.R^2^ = 0.25, *p* < 0.001, f^2^ = 0.38				
Adiposity	**− **0.001	0.01	**− **0.05	0.96
Age	**0.06**	**0.02**	**3.55**	** < 0.001**
Testosterone LOG	**0.61**	**0.18**	**3.35**	**0.001**
AST/ALT LOG	0.38	0.24	1.54	0.13

Bolded values are significant at *p* < 0.05

As previous studies have failed to demonstrate a relationship between testosterone and perceived physical attractiveness or masculinity (Kordsmeyer et al., 2019; Lefevre et al., 2013; Neave et al., 2003; Peters et al., 2008; Arnocky & Davis, 2024), we also conducted regression models excluding testosterone. Excluding testosterone from the models with physical attractiveness as a dependent variable did not change the fit of any of the models (Model 1: ΔAIC = 0; Model 2: ΔAIC = 1; Model 3: ΔAIC = 1; Model 4: ΔAIC = 1; Model 5: ΔAIC = 0). The models also did not improve in terms of variance explained (Model 1: ΔR2 = 0.01, *p* = 0.28; Model 2: ΔR2 = 0.01, *p* = 0.30; Model 3: ΔR2 = 0.01, *p* = 0.32; Model 4: ΔR2 = 0.01, *p* = 0.24; Model 5: ΔR2 = 0.01, *p* = 0.23). However, including testosterone in the analyses with perceived masculinity as the dependent variable improved the fit of all models (Model 1: ΔAIC = 10; Model 2: ΔAIC = 9; Model 3: ΔAIC = 10; Model 4: ΔAIC = 10; Model 5: ΔAIC = 9). Additionally, the models that included testosterone levels explained more variance (Model 1: ΔR2 = 0.09, *p* < 0.001; Model 2: ΔR2 = 0.08, *p* = 0.002; Model 3: ΔR2 = 0.09, *p* < 0.001; Model 4: ΔR2 = 0.09,* p* < 0.001; Model 5: ΔR2 = 0.08, *p* = 0.001).

Mediation analyses showed that the relationship between markers of cardiometabolic health (except for cardiometabolic risk score) and physical attractiveness was mediated by adiposity (Table 6). There was no mediation effect for the relationship between physical attractiveness and markers of cardiometabolic health by age (Indirect effects: Model 1: b < 0.001, *p* = 0.96; Model 2: b = 0.04, *p* = 0.55; Model 3: b < -0.001, *p* = 0.99; Model 4: b = 0.01, *p* = 0.83; Model 5: b =  − 0.05, *p* = 0.65; direct and total effects are reported in the Supplementary materials—Table S1) or testosterone levels (Indirect effects: Model 1: b =  − 0.08, *p* = 0.15; Model 2: b = -0.35, *p* = 0.16; Model 3: b = -0.07 *p* = 0.29; Model 4: b = -0.20, *p* = 0.13; Model 5: b = 0.36, *p* = 0.15; direct and total effects are reported in the Supplementary materials—Table S2).

**Table 6:** Results for the mediation analyses for the mediating effect of adiposity on the relationship between facial attractiveness and selected cardiometabolic risk markers (N = 105)

	Estimate (b)	SE	95% Confidence level	*p*
Model 1: Dependent variable: facial attractiveness; Predictor: Cholesterol-triglyceride index; Mediator: Adiposity				
Indirect effect	**− 0.19**	**0.09**	**[− 0.36;− 0.03]**	**0.03**
Direct effect	**− **0.19	0.16	[**− **0.55; 0.09]	0.23
Total effect	**− 0.38**	**0.14**	**[− 0.67;− 0.13]**	**0.007**
Model 2: Dependent variable: facial attractiveness; Predictor: Triglycerides level; Mediator: Adiposity				
Indirect effect	**− 0.002**	**0.001**	**[− 0.004;− 0.001]**	**0.02**
Direct effect	**− **0.001	0.001	[**− **0.004;0.001]	0.26
Total effect	**− 0.003**	**0.001**	**[− 0.006;− 0.001]**	**0.002**
Model 3: Dependent variable: facial attractiveness; Predictor: Cardiometabolic risk score; Mediator: Adiposity				
Indirect effect	**− **0.18	0.14	[**− **0.49;0.08]	0.21
Direct effect	**− **0.29	0.20	[**− **0.72;0.09]	0.15
Total effect	**− 0.47**	**0.14**	**[− 0.75;− 0.23]**	** < 0.001**
Model 4: Dependent variable: facial attractiveness; Predictor: HOMA-IR; Mediator: Adiposity				
Indirect effect	**− 0.56**	**0.23**	**[− 1.18;− 0.06]**	**0.05**
Direct effect	**− **0.38	0.47	[**− **1.32;0.52]	0.41
Total effect	**− 0.94**	**0.36**	**[− 1.68;− 0.26]**	**0.008**
Model 5: Dependent variable: facial attractiveness: Predictor: AST/ALT; Mediator: Adiposity				
Indirect effect	**1.02**	**0.46**	**[0.18;1.99]**	**0.03**
Direct effect	0.50	0.60	[**− **0.66;1.67]	0.40
Total effect	**1.53**	**0.49**	**[0.59;2.50]**	**0.002**

Bolded values are significant at *p* < 0.05

Mediation analyses showed that the relationship between perceived facial masculinity and cardiometabolic health was mediated by testosterone (Table 7). There was no mediation effect for the relationship between perceived facial masculinity and markers of cardiometabolic health by adiposity (Indirect effects: Model 1: b = -0.14, *p* = 0.12; Model 2: b = -0.32, *p* = 0.18; Model 3: b = -0.18,* p* = 0.22; Model 4: b = -0.37, *p* = 0.17; Model 5: b = 0.42, *p* = 0.34; direct and total effects are reported in the Supplementary materials—Table S3) or age (Indirect effects: Model 1: b =  − 0.003, *p* = 0.94; Model 2: b = -0.11, *p* = 0.32; Model 3: b < 0.001, *p* = 0.99; Model 4: b = -0.04, *p* = 0.72; Model 5: b = 0.13, *p* = 0.48; direct and total effects are reported in the Supplementary materials—Table S4).

**Table 7:** Results for the mediation analyses for the mediating effect of testosterone on the relationship between facial masculinity and selected cardiometabolic risk markers (N = 105)

	Estimate (b)	SE	95% Confidence level	*p*
Model 1: Dependent variable: facial masculinity; Predictor: Cholesterol-triglyceride index; Mediator: Testosterone				
Indirect effect	**− 0.16**	**0.07**	**[− 0.04;− 2.23]**	**0.03**
Direct effect	**− **0.03	0.12	[**− **0.28;0.18]	0.79
Total effect	**− **0.19	0.14	[**− **0.48;0.07]	0.17
Model 2: Dependent variable: facial masculinity; Predictor: Triglycerides level; Mediator: Testosterone				
Indirect effect	**− 0.67**	**0.25**	**[− 1.20;− 0.22]**	**0.008**
Direct effect	**− **0.23	0.37	[**− **1.00;0.46]	0.53
Total effect	**− 0.90**	**0.38**	**[− 1.65;− 0.17]**	**0.02**
Model 3: Dependent variable: facial masculinity; Predictor: Cardiometabolic risk score; Mediator: Testosterone				
Indirect effect	**− 0.18**	**0.07**	**[− 0.34;− 0.06]**	**0.01**
Direct effect	**− **0.05	0.11	[**− **0.29;0.15]	0.63
Total effect	**− 0.24**	**0.12**	**[− 0.49;− 0.01]**	**0.05**
Model 4: Dependent variable: facial masculinity; Predictor: HOMA-IR; Mediator: Testosterone				
Indirect effect	**− 0.36**	**0.15**	**[− 0.72;− 0.12]**	**0.02**
Direct effect	**− **0.26	0.27	[**− **0.80;0.27]	0.34
Total effect	**− 0.62**	**0.30**	**[− 1.23;− 0.05]**	**0.04**
Model 5: Dependent variable: facial masculinity: Predictor: AST/ALT; Mediator: Testosterone				
Indirect effect	**0.38**	**0.12**	**[0.16;0.62]**	**0.001**
Direct effect	**1.52**	**1.52**	**[0.63;2.29]**	** < 0.001**
Total effect	**0.96**	**0.96**	**[0.06;1.79]**	**0.03**

Bolded values are significant at *p* < 0.05

## Discussion

The results of our study showed that facial attractiveness and masculinity in healthy adult men were negatively related to adiposity, HOMA-IR, triglycerides level, and cardiometabolic risk score. Additionally, we confirmed the positive relation of perceived facial masculinity and age, total testosterone level, AST/ALT ratio, and HDL cholesterol. Facial attractiveness showed similar associations, except for age. However, none of these relationships remained significant when controlling for age, adiposity, and testosterone level. After adjusting for these factors, facial attractiveness was negatively associated only with adiposity, while facial masculinity was positively associated with age and testosterone levels. These findings align with previous research indicating that men’s facial appearance conveys information about age (Boothroyd et al., 2005), adiposity (Coetzee et al., 2007), and testosterone levels (Penton-Voak & Chen, 2004) but not necessarily about health (Cai et al., 2019), at least in younger, healthy men. However, it is important to emphasize that previous studies have yielded mixed results, with many failing to find a significant association between perceived attractiveness and factors such as testosterone levels (Kordsmeyer et al., 2019; Peters et al., 2008; Swaddle & Reierson, 2002).

Men’s facial attractiveness and masculinity were negatively associated with triglyceride levels and positively associated with the beneficial HDL fraction of the lipid profile. However, these relationships were not significant when controlling for age, adiposity, and testosterone levels, suggesting that these factors mediate the relationship between facial appearance and lipid profile in men. Previous research showed that facial attractiveness is negatively correlated with TG levels (when controlled for BMI, estradiol, and testosterone) in healthy women of reproductive age (Żelaźniewicz et al., 2020a). To our knowledge, no other studies have explored the relationship between facial attractiveness/masculinity and lipid profile. The sex difference observed in both our study and previous research may be explained by the fact that although abnormal lipid profiles generally have more detrimental effects on men (Goossens et al., 2021; Kamon et al., 2022), increased TG levels are more harmful for women compared to men (Castelli, 1992), and thus might be more important for a woman’s biological condition. In the case of healthy adult men, there is likely no direct relationship between attractiveness/masculinity and lipid profile due to the different effects of sex hormones on lipid profiles than in women. On the other hand, despite the lack of a relationship between masculinity and lipid profile, testosterone was negatively correlated with triglycerides and the cholesterol-triglyceride index and positively correlated with HDL cholesterol. This is consistent with studies suggesting that lower testosterone levels are associated with a worsened lipid profile in men (Grandys et al., 2021). Thus, facial appearance may reflect androgen levels, which are a more general marker of biological condition, but is not a good cue for lipid profiles in men.

Similarly, we found a negative relationship between facial attractiveness and masculinity and the insulin resistance index (HOMA-IR). However, this relationship became non-significant when controlling for age, testosterone levels, or adiposity. We also found no relationship between facial appearance and HbA1c levels, a marker of long-term glycemia. On the other hand, testosterone levels were negatively related to adiposity and HOMA-IR. So far, only a few studies have addressed the relationship between facial appearance and glycemia and most of them concern the positive association between glucose level and perceived facial age in elders and diabetics (Noordam et al., 2013). In addition to perceived age, hyperglycemia and insulin resistance are linked to decreased testosterone levels in men (Lutz et al., 2019), which may, in turn, be associated with reduced attractiveness and masculinity. However, in our previous study conducted on the same group of men, this effect was not confirmed (Żelaźniewicz et al., 2022). Similarly, no relationship was found between glycemia markers (HOMA-IR, HbA1c, and C-peptide levels) and facial appearance in young women (Żelaźniewicz et al., 2020a). This suggests that the relationship between glycemia level and facial appearance may be rather detectable in elders or individuals with diabetes. Interestingly, studies on the effect of high-glycemic meal consumption on facial attractiveness show contradictory results. Visine et al. (2024) demonstrated that the immediate consumption of a high-glycemic meal, which induces hyperglycemia, decreased facial attractiveness in both men and women, while Berticat et al. (2020) found that individuals with the highest between-meal glycemic loads were preferred by opposite-sex raters. Therefore, it remains unclear whether glycemia contributes to facial appearance in healthy young individuals.

The AST to ALT ratio (De Ritis ratio) positively correlated with facial attractiveness and masculinity, but this relationship was non-significant when controlled for age, testosterone, and adiposity. De Ritis ratio was also positively correlated with testosterone level. Previous research found no association between facial attractiveness and liver enzyme levels in healthy women of reproductive age (Żelaźniewicz et al., 2020a). To our knowledge, there is no other research connecting facial appearance with liver function markers. It is known that liver malfunction may manifest in the appearance of the skin through pigmentary changes, spider angiomas, “paper money” skin, or *xanthelasma*s. Studies show that skin surface topography and coloration can impact perceptions of attractiveness and age in both sexes (Fink et al., 2018; Samson et al., 2010). A lower De Ritis ratio is related to some health problems like hepatic steatosis—NAFLD (Yang et al., 2019) or metabolic syndrome (Yadav et al., 2016). Interestingly, men are at higher risk of NAFLD development compared to women (Balakrishnan et al., 2021), which may explain the lack of a similar relationship in women found in previous research (Żelaźniewicz et al., 2020a).

The results of our study suggest that men’s facial attractiveness and masculinity are primarily predicted by body adiposity and testosterone levels. Adiposity mediated the relationship between cardiometabolic health and perceived attractiveness, while testosterone mediated the relationship between cardiometabolic health and perceived masculinity. These findings align with Foo et al. (2017), who demonstrated that facial adiposity is a stronger predictor of attractiveness than sexual dimorphism, averageness, or symmetry. Similarly, Coetzee et al. (2009) found that perceived facial adiposity predicts attractiveness and health, correlating with cardiovascular health and reported infection frequency. A meta-analysis by De Jager et al. (2018) showed that people could reliably estimate BMI from facial cues alone, which is important because obesity is recognized as a risk factor for several diseases (Sarma et al., 2021) and it shortens life expectancy by up to 20 years (Fontaine et al., 2003). Interestingly, recent studies indicate that perceived facial adiposity might be a better indicator of health outcomes than traditional measures of obesity, such as BMI, percentage body fat, or waist girth (Coetzee et al., 2009; Tafeit et al., 2000). Furthermore, similarly to adiposity, T level is also a general measure of a man’s biological condition. Low testosterone is associated with a range of long-term health impairments, including increased risk of dyslipidemia (Haring et al., 2011), diabetes (Laaksonen et al., 2004), decreased cardiometabolic health, and higher mortality rates (Holmboe et al., 2018). Testosterone is also positively correlated with muscle strength, dominance, and aggression, all of which increase the likelihood of winning in intrasexual competition (Archer, 1991; Archer, 2006; Chiu et al., 2020). While some studies show no correlation between testosterone levels and attractiveness (Swaddle & Reierson, 2002), and the relationship between testosterone and facial masculinity is mixed (Kordsmeyer et al., 2019; Lefevre et al., 2013; Neave et al., 2003; Pound et al., 2009; Roney et al., 2006), others suggest that women prefer masculine faces in a mating context (Docherty et al., 2020; Ekrami et al., 2021). Thus, we propose that while facial appearance may not directly indicate cardiometabolic health, it may serve as a marker of adiposity and testosterone levels, which, in healthy adult men, may be more crucial for biological fitness than specific cardiometabolic health measures.

We also found a positive correlation between perceived facial masculinity (but not attractiveness) and age, which aligns with the previous studies (Boothroyd et al., 2005, Alharbi et al., 2021; Coetzee et al., 2005). An increase in facial masculinity with age may result from morphological changes associated with maturation, such as redistribution of subcutaneous fullness, resulting in bony structures becoming more prominent, changes in facial proportion, or subtle changes linked with aging (Coleman & Grover, 2006; Ilankovan, 2014) that may contribute to enhanced masculinity.

Interpreting these results requires consideration of the study's limitations. First, no causal relationships can be inferred due to its cross-sectional design. Second, cardiometabolic risk markers were assessed only once. Despite a rigorous study protocol, we cannot rule out the possibility of intra-individual variability in the measured biomarkers. Future studies should incorporate repeated measurements of cardiometabolic risk markers to address this limitation.

In summary, the results of our study suggest that in a sample of healthy adult men, adiposity and testosterone level may contribute to the relationship between appearance characteristics and broader health status (including cardiometabolic health) (Ameratunga et al., 2023; Hansen et al., 2023). The hormonal system, body composition, and overall cardiometabolic health are a network of interconnected factors that simultaneously affect appearance and broadly understood “biological condition” (Kaur & Werstuck, 2021; Mutie et al., 2023). As a result, testosterone and body adiposity may account for a significant portion of the variance in cardiometabolic functioning, potentially mediating the relationship between specific cardiometabolic markers and physical appearance. It is plausible that sexual selection favored sensitivity to broader morphological cues of health or fertility, such as higher body adiposity or reduced testosterone, rather than for any specific physiology-dependent health problems. Nonetheless, it is important to note that these preferences may also be shaped by environmental and cultural factors. For instance, in populations with scarce food or where modern media exposure is limited, higher adiposity may be valued as a sign of resource abundance or resilience (Tovée et al., 2006; Boothroyd et al., 2020). Our findings, also align more closely with the “bad gene avoidance” perspective rather than the “hunting for good genes” hypothesis, suggesting that individuals may be more inclined to avoid potential mates who display cues of compromised health, rather than actively seeking out those with cues of the best possible health, a strategy that may serve to increase the pool of acceptable mates. Male facial appearance may therefore function to help distinguish individuals with generally poorer health based on broad health-related cues, rather than to single out the one “healthiest” phenotype.

## Supplementary Information

Below is the link to the electronic supplementary material.

Supplementary file1 (DOCX 268 KB)

Supplementary file2 (XLSX 29 KB)

## Data Availability

Data are available as supplementary material.
