# Supplementary material for: Facial Appearance and Markers of Cardiometabolic Risk in Healthy Adult Men
Source: Arch Sex Behav. 2025 Aug 20;54(7):2491–504. doi: 10.1007/s10508-025-03205-3 (PMC12457508; doi:10.1007/s10508-025-03205-3)
Supplement: Supplementary file 1 — Supplementary file1 (DOCX 268 KB) [file 10508_2025_3205_MOESM1_ESM.docx]

Supplementary materials

Fig. S1. Correlation between facial masculinity and attractiveness and HOMA-IR levels (N=105)

Fig. S2. Correlation between facial masculinity and attractiveness and triglycerides levels (N=105)

Fig. S3. Correlation between facial masculinity and attractiveness and cholesterol-to-triglycerides levels (N=105)

Fig. S4. Correlation between facial masculinity and attractiveness and cardiometabolic risk score (N=105)

Fig. S5. Correlation between facial masculinity and attractiveness and AST/ALT ratio (N=105)

**Table S1.** Results for the mediation analyses for the mediating effect of age on the relationship between facial attractiveness and selected cardiometabolic risk markers (N=105). Bolded values are significant at *p*<0.05.

|  | Estimate (b) | | SE | 95% Confidence level | *p* |
| --- | --- | --- | --- | --- | --- |
| ***Model 1: Dependent variable: facial attractiveness; Predictor: Cholesterol-triglyceride index; Mediator: Age*** | | | | | |
| Indirect effect | | <0.001 | 0.02 | [-0.03;0.04] | 0.96 |
| **Direct effect** | | **-0.38** | **0.14** | **[-0.68;-0.13]** | **0.008** |
| **Total effect** | | **-0.38** | **0.14** | **[-0.67;-0.13]** | **0.006** |
| ***Model 2: Dependent variable: facial attractiveness; Predictor: Triglycerides level; Mediator: Age*** | | | | | |
| Indirect effect | | 0.04 | 0.07 | [-0.06;0.22] | 0.55 |
| **Direct effect** | | **-1.03** | **0.38** | **[-1.76;-0.30]** | **0.006** |
| **Total effect** | | **-0.98** | **0.37** | **[-1.72;-0.26]** | **0.008** |
| ***Model 3: Dependent variable: facial attractiveness; Predictor: Cardiometabolic risk score; Mediator: Age*** | | | | | |
| Indirect effect | | <-0.001 | 0.02 | [-0.04;0.05] | 0.99 |
| **Direct effect** | | **-0.47** | **0.14** | **[-0.76;-0.23]** | **<0.001** |
| **Total effect** | | **-0.47** | **0.13** | **[-0.76;-0.23]** | **<0.001** |
| ***Model 4: Dependent variable: facial attractiveness; Predictor: HOMA-IR; Mediator: Age*** | | | | | |
| Indirect effect | | 0.01 | 0.06 | [-0.07;0.18] | 0.84 |
| **Direct effect** | | **-0.96** | **0.37** | **[-1.69;-0.26]** | **0.009** |
| **Total effect** | | **-0.94** | **0.36** | **[-1.65;-0.26]** | **0.008** |
| ***Model 5: Dependent variable: facial attractiveness: Predictor: AST/ALT; Mediator: Age*** | | | | | |
| Indirect effect | | -0.05 | 0.10 | [-0.32;0.11] | 0.65 |
| **Direct effect** | | **1.57** | **0.51** | **[0.62;2.63]** | **0.002** |
| **Total effect** | | **1.53** | **0.50** | **[0.59;2.53]** | **0.002** |

**Table S2.** Results for the mediation analyses for the mediating effect of testosterone on the relationship between facial attractiveness and selected cardiometabolic risk markers (N=105). Bolded values are significant at *p*<0.05.

|  | Estimate (b) | | SE | 95% Confidence level | *p* |
| --- | --- | --- | --- | --- | --- |
| ***Model 1: Dependent variable: facial attractiveness; Predictor: Cholesterol-triglyceride index; Mediator: Testosterone*** | | | | | |
| Indirect effect | | -0.08 | 0.06 | [-0.21;0.01] | 0.15 |
| **Direct effect** | | **-0.30** | **0.15** | **[-0.61;-0.03]** | **0.04** |
| **Total effect** | | **-0.38** | **0.14** | **[-0.67;-0.12]** | **0.007** |
| ***Model 2: Dependent variable: facial attractiveness; Predictor: Triglycerides level; Mediator: Testosterone*** | | | | | |
| Indirect effect | | -0.35 | 0.25 | [-0.85;0.11] | 0.16 |
| Direct effect | | -0.64 | 0.44 | [-1.48;0.27] | 0.14 |
| **Total effect** | | **-0.98** | **0.38** | **[-1.72;-0.20]** | **0.01** |
| ***Model 3: Dependent variable: facial attractiveness; Predictor: Cardiometabolic risk score; Mediator: Testosterone*** | | | | | |
| Indirect effect | | -0.07 | 0.06 | [-0.21;0.04] | 0.29 |
| **Direct effect** | | **-0.40** | **0.15** | **[-0.71;-0.11]** | **0.008** |
| **Total effect** | | **-0.47** | **0.14** | **[-0.75;-0.22]** | **<0.001** |
| ***Model 4: Dependent variable: facial attractiveness; Predictor: HOMA-IR; Mediator: Testosterone*** | | | | | |
| Indirect effect | | -0.20 | 0.13 | [-0.51;0.01] | 0.13 |
| **Direct effect** | | **-0.75** | **0.37** | **[-0.47;-0.03]** | **0.04** |
| **Total effect** | | **-0.94** | **0.36** | **[-1.66;-0.25]** | **0.008** |
| ***Model 5: Dependent variable: facial attractiveness: Predictor: AST/ALT; Mediator: Testosterone*** | | | | | |
| Indirect effect | | 0.36 | 0.25 | [-0.02;0.94] | 0.15 |
| **Direct effect** | | **1.17** | **0.54** | **[0.11;2.21]** | **0.03** |
| **Total effect** | | **1.53** | **0.49** | **[0.59;2.51]** | **0.002** |

**Table S3.** Results for the mediation analyses for the mediating effect of adiposity on the relationship between facial masculinity and selected cardiometabolic risk markers (N=105). Bolded values are significant at *p*<0.05.

|  | Estimate (b) | | SE | 95% Confidence level | *p* |
| --- | --- | --- | --- | --- | --- |
| ***Model 1: Dependent variable: facial masculinity; Predictor: Cholesterol-triglyceride index; Mediator: Adiposity*** | | | | | |
| Indirect effect | | -0.14 | 0.09 | [-0.33;0.01] | 0.12 |
| Direct effect | | -0.05 | 0.15 | [-0.36;0.23] | 0.73 |
| Total effect | | -0.19 | 0.14 | [-0.48;0.06] | 0.17 |
| ***Model 2: Dependent variable: facial masculinity; Predictor: Triglycerides level; Mediator: Adiposity*** | | | | | |
| Indirect effect | | -0.32 | 0.23 | [-0.80;0.13] | 0.18 |
| Direct effect | | -0.59 | 0.38 | [-1.33;0.17] | 0.12 |
| **Total effect** | | **-0.90** | **0.38** | **[-1.68;-0.18]** | **0.02** |
| ***Model 3: Dependent variable: facial masculinity; Predictor: Cardiometabolic risk score; Mediator: Adiposity*** | | | | | |
| Indirect effect | | -0.18 | 0.15 | [-0.50;0.07] | 0.22 |
| Direct effect | | -0.06 | 0.18 | [-0.41;0.30] | 0.75 |
| Total effect | | -0.24 | 0.12 | [-0.50;-0.01] | 0.06 |
| ***Model 4: Dependent variable: facial masculinity; Predictor: HOMA-IR; Mediator: Adiposity*** | | | | | |
| Indirect effect | | -0.37 | 0.26 | [-0.95;0.10] | 0.17 |
| Direct effect | | -0.26 | 0.35 | [-0.94;0.44] | 0.47 |
| **Total effect** | | **-0.62** | **0.30** | **[-1.22;-0.06]** | **0.04** |
| ***Model 5: Dependent variable: facial masculinity: Predictor: AST/ALT; Mediator: Adiposity*** | | | | | |
| Indirect effect | | 0.43 | 0.44 | [-0.36;1.37] | 0.34 |
| **Direct effect** | | **1.11** | **0.57** | **[-0.07;2.20]** | **0.05** |
| **Total effect** | | **1.53** | **0.48** | **[0.63;2.50]** | **0.001** |

**Table S4.** Results for the mediation analyses for the mediating effect of age on the relationship between facial masculinity and selected cardiometabolic risk markers (N=105). Bolded values are significant at *p*<0.05.

|  | Estimate (b) | | SE | 95% Confidence level | *p* |
| --- | --- | --- | --- | --- | --- |
| ***Model 1: Dependent variable: facial masculinity; Predictor: Cholesterol-triglyceride index; Mediator: Age*** | | | | | |
| Indirect effect | | -0.003 | 0.04 | [-0.09;0.07] | 0.94 |
| Direct effect | | -0.19 | 0.12 | [-0.45;0.03] | 0.12 |
| Total effect | | -0.19 | 0.14 | [-0.49;0.05] | 0.17 |
| ***Model 2: Dependent variable: facial masculinity; Predictor: Triglycerides level; Mediator: Age*** | | | | | |
| Indirect effect | | -0.11 | 0.12 | [-0.37;0.09] | 0.32 |
| **Direct effect** | | **-0.79** | **0.35** | **[-1.47;-0.10]** | **0.02** |
| **Total effect** | | **-0.90** | **0.39** | **[-1.67;-0.15]** | **0.02** |
| ***Model 3: Dependent variable: facial masculinity; Predictor: Cardiometabolic risk score; Mediator: Age*** | | | | | |
| Indirect effect | | <0.001 | 0.04 | [-0.09;0.08] | 0.99 |
| **Direct effect** | | **-0.24** | **0.11** | **[-0.47;-0.04]** | **0.03** |
| **Total effect** | | **-0.24** | **0.12** | **[-0.50;-0.02]** | **0.05** |
| ***Model 4: Dependent variable: facial masculinity; Predictor: HOMA-IR; Mediator: Age*** | | | | | |
| Indirect effect | | -0.04 | 0.11 | [-0.27;0.19] | 0.72 |
| **Direct effect** | | **-0.58** | **0.27** | **[-1.15;-0.09]** | **0.03** |
| **Total effect** | | **-0.62** | **0.30** | **[-1.25;-0.06]** | **0.04** |
| ***Model 5: Dependent variable: facial masculinity: Predictor: AST/ALT; Mediator: Age*** | | | | | |
| Indirect effect | | 0.13 | 0.18 | [-0.22;0.51] | 0.48 |
| **Direct effect** | | **1.40** | **0.44** | **[0.54;2.29]** | **0.002** |
| **Total effect** | | **1.53** | **0.48** | **[0.60;2.49]** | **0.001** |
